# Evaluation of clinicians’ knowledge and practice regarding pharmacotherapy of Non-Hodgkin’s lymphoma: A multi-center study in Yemen

**DOI:** 10.1371/journal.pone.0304209

**Published:** 2024-06-05

**Authors:** Mohammed Mohammed Battah, Hadzliana Zainal, Doa’a Anwar Ibrahim, Nur Hafzan Binti Md Hanafiah, Syed Azhar Syed Sulaiman

**Affiliations:** 1 Discipline of Clinical Pharmacy, School of Pharmaceutical Sciences, Universiti Sains Malaysia, Penang, Malaysia; 2 Department of Clinical Pharmacy and Pharmacy Practice, Faculty of Pharmacy, University of Science and Technology, Sana’a, Yemen; Bursa Ali Osman Sonmez Oncology Hospital, TURKEY

## Abstract

Non-Hodgkin lymphoma (NHL) is a hematological malignancy that requires effective pharmacotherapy for optimal management. There is limited information regarding Yemeni clinicians’ knowledge and practice of NHL pharmacotherapy. This study aims to assess the knowledge and practice of physicians and nurses in Yemen regarding pharmacotherapy of NHL. A cross-sectional study was conducted in Sana’a, Yemen, from January 1, 2022, to January 31, 2023. Two self-administrated and validated questionnaires were distributed to 99 physicians and 164 nurses involved in pharmacotherapy for NHL in different oncology centers and units across Yemen. Convenience samples were used to recruit participants. A binary logistic regression analysis was performed to identify factors associated with nurses’ and physicians’ knowledge and practice. The correlation coefficient was used to examine the relationship between knowledge and practice. A total of 77 physicians and 105 nurses completed the questionnaires. The results showed that 54.3% of nurses and 66.2% of physicians had poor knowledge of NHL pharmacotherapy. In terms of practice, 83.8% of nurses and 75.3% of physicians exhibited poor practice regarding NHL pharmacotherapy. Multivariable logistic regression analysis identified that nurses who received sufficient information about chemotherapy displayed a significant association with good knowledge, while nurses working in the chemotherapy administration department were significant predictors of good practice. Among physicians, those working in the National Oncology Center (NOC) in Sana’a demonstrated good practice. Correlation analysis revealed a positive relationship between nurses’ knowledge and their practice. The study’s results confirm deficiencies in knowledge and practice of pharmacotherapy for NHL among physicians and nurses in Yemen. Efforts should be made to enhance their understanding of treatment guidelines and to improve patient care. Improvement in educational programs and training opportunities may contribute to improving patient outcomes in the management of NHL.

## Introduction

Non-Hodgkin lymphoma (NHL) is a hematologic malignancy that arises from immune cells in lymphoid and other lymphoid tissues outside the nodes [1]. It is the most common type of lymphoma, accounting for about 85% of all lymphomas [2]. NHL can be classified into two main prognostic groups: aggressive and indolent NHLs [3]. It is further classified, according to the World Health Organization (WHO), into T-cell, NK-cell, and B-cell subtypes. Diffuse large B-cell lymphoma (DLBCL) is the most common subtype of all NHLs [4]. NHL treatment strategies vary based on several factors, including the specific subtype, disease stage, affected sites, and individual patient characteristics such as age and comorbidities. These strategies may include chemotherapy, radiation therapy, or a combination of these approaches and may involve only patient observation [5, 6].

The most common pharmacotherapeutic agents used in the treatment of NHL include cyclophosphamide, bleomycin, doxorubicin, purine analogs, etoposide, methotrexate, vincristine, and corticosteroids. However, Rituximab-based regimens have shown encouraging outcomes in terms of overall survival rates [5]. On the other hand, radiation therapy is often reserved as a palliative measure to manage extensive local disease or as a consolidation strategy post-chemotherapy in patients with extranodal large masses [5, 6]. The pharmacotherapy process is an essential part of patient care, as it involves the use of medications to manage diseases and symptoms [7]. However, the complexity of this process introduces the potential for medication errors [8], which most commonly occur in physicians’ prescriptions (46%) and nurses’ administration (41%) [9, 10]. This underscores the importance of the role of clinicians, including physicians and nurses, in prescribing and administering medications, as they have significant impacts on pharmacotherapy outcomes and patients’ recovery [7].

In cancer pharmacotherapy, physicians and nurses are of utmost importance in the successful implementation of effective anti-cancer measures, particularly in the early detection and management of cases [11]. Despite this, their knowledge and awareness about pharmacotherapy of cancer, if insufficient, can lead to poor clinical practices, negative attitudes, and, ultimately, worse patient outcomes. This inadequate knowledge also makes it challenging to develop effective cancer prevention and control strategies [11]. Moreover, clinicians’ adherence to NHL pharmacotherapy guidelines is more likely to provide high-quality patient care. However, their lack of adherence to guidelines can lead to suboptimal care, underscoring the need for interventions to improve adherence and the quality of care [12].

While few studies have been conducted worldwide to assess physicians’ knowledge and practices in the field of cancer, they have focused primarily on pain control and palliative care areas [13, 14]. Studies concerning nurses, in particular, have explored their knowledge and practices regarding the administration of chemotherapy [15, 16]. This literature, however, did not address the specific complexities of NHL pharmacotherapy, which represents a significant gap in the current body of literature. According to the WHO: Globocan 2022 in the Yemeni context, NHL accounted for 851 new cases in Yemen, ranking sixth in terms of incidence among both sexes and third among males. It represented 5.2% of all new cancer cases with a cumulative risk of 0.35%. The number of deaths due to NHL was 585 cases, representing 4.9%, ranking as the ninth-highest number of fatalities, with a cumulative risk of 0.25. The 5-year prevalence of NHL cases was 1,829, representing 5.8% of all prevalent cancer cases [17]. Particularly, in Aden Governorate, in Aden Governorate, NHL emerged as the predominant type of malignant hematological tumors in both 2010 and 2013, according to data from the National Oncology Center (NOC) [18, 19]. Lymphomas comprised 9.8% of all cancer cases, with NHL representing 65% of all lymphoma patients [19].

Despite the alarming prevalence of NHL in Yemen, there is a lack of research focusing on the knowledge and practices of Yemeni clinicians regarding the pharmacotherapy of NHL. Therefore, this study aims to fill this research gap and to assess the knowledge and practice of physicians and nurses regarding pharmacotherapy for NHL in Yemen. Also, to assess the relationship between the knowledge and practice of physicians and nurses regarding pharmacotherapy for NHL.

## Methods

### Ethical approval

The Ethical Committee of the Medical Research, University of Sciences and Technology, Sana’a, Yemen, approved this project, EAC/UST201 (this study is part of a project about Medication Use Evaluation for Non-Hodgkin Lymphoma in Yemen). The ethical committee approved the use of verbal informed consent in this study, as the participants’ identities were kept entirely anonymous, ensuring their privacy, and the study did not involve any risk to the participants. Therefore, clinicians who gave verbal consent and agreed to participate in the study were included. Additionally, all participants received thorough explanations of the study’s aims and objectives, and they were allowed to ask research-related questions.

### Study design and setting

A cross-sectional study was conducted to investigate the pharmacotherapy of NHL in Yemen. Structured and validated questionnaires were used to collect data from physicians and nurses involved in the treatment of NHL across various oncology centers and units. Face-to-face interviews were conducted with participants in eight different regions in Yemen, including Sana’a Governorate (the NOC in Al-Jomhouri Teaching Hospital and the Oncology Unit in Al-Kuwait Hospital), Aden Governorate (the NOC in Al-Sadaqah Hospital), Hadramout Governorate (the NOCs in Al-Mukalla, and Hadramout Valley and Desert (Seiyun), Taiz Governorate (Al-Amal Oncology Center), and Ibb and Al-Hudaidah Governorates (Al-Amal Oncology Units). These areas were selected to ensure the representation of oncology centers or units in Yemen’s north and south governorates. The study spanned 13 months, from January 1, 2022, to January 31, 2023.

### Study population

The study included all oncology physicians and nurses working in the oncology centers and units in Yemen. Since the overall number of officially registered oncologists in Yemen is few [20], subjects were enrolled in this study using the entire population, as all physicians and nurses in all oncology centers and units were invited to participate in the study. Those involved in NHL pharmacotherapy and who agreed to participate were included in this study, while physicians and nurses who refused to participate were excluded. The total number of physicians in all large oncology centers in Yemen was 83, and 136 nurses [21]. However, to broaden the scope of our study, we also collected data from different oncology units in Yemen. After getting the information from these oncology units by personal communication, we found that the actual number across all oncology units in Yemen was 16 physicians and 28 nurses. Taking these additional statistics into account, we found that the final total number across all oncology centers and units in all regions in Yemen was 99 physicians and 164 nurses.

### Sample size calculation and participants

Convenience sampling was used to recruit participants for our study [11, 22]. Given the descriptive nature of our study and the rarity of NHL, as well as the limited available information on the subject, the sample size was determined by including all physicians and nurses from oncology centers and units [11]. The targeted physicians were categorized into consultants (those who have a subspecialty), specialists (physicians who completed four or five years of residency program), residents (physicians enrolled in a four or 5-year residency program), and general physicians (GP; licensed physicians who are graduated from an accredited medical school without being enrolled into a residency program).

### Study instrument

Two self-administered questionnaires were designed: one for physicians and another for nurses. The questionnaires were developed based on the guidelines’ recommendations, experts’ opinions, and previously published literature [23–26]. Each questionnaire comprises three sections (S1 and S2 Files): the first included sociodemographic characteristics, the second contained questions concerning knowledge, and the third included questions concerning practice regarding the pharmacotherapy of NHL.

For physicians, section 1 contained data about age, gender, marital status, specialty and subspecialty, experience years, and working place. Section 2 included eighteen questions designed to assess the participants’ overall knowledge regarding the pharmacotherapy of NHL with three possible responses (agree, disagree, don’t know). Section 3 contained 16 questions that evaluated the physicians’ practices for pharmacotherapy of NHL in Yemen. Eleven questions with responses were measured on a 5-point Likert scale: never, rarely, sometimes, often, always, and the 6^th^ response was “not applicable”.

For Nurses, section 1 contained data about age, gender, marital status, experience years, professional qualification, and working ward. Furthermore, this section included a question about whether the participants had any chemotherapy training program in the past five years and how many programs if the answer was yes [27]. The last question in this section was about the adequacy of the information the participants had received on chemotherapy during their studies. Section 2 included fifteen questions designed to assess the participants’ overall knowledge regarding the pharmacotherapy of NHL with three possible responses (agree, disagree, don’t know). Section 3 contained 16 questions that evaluated the nurses’ practices regarding pharmacotherapy of NHL, with responses being measured on a 5-point Likert scale: never, rarely, sometimes, often, always, and the 6^th^ response was “not applicable”.

### Scoring system

The correct answer received one point, while the incorrect or “I do not know” answers were given zero points. Therefore, the highest possible score for nurses’ knowledge is 15, and the lowest is 0. Bloom’s cut-off of 80% was adopted to categorize knowledge, and a score of ≥12 was considered good knowledge. For physicians, the total knowledge score ranged from 0 to 18. The total knowledge score was classified using Bloom’s cut-off point; a score of ≥14.4 was considered good knowledge (14.4–18, 80–100%) [28]. In the assessment of practices, a scoring system ranging from 1 to 5 was used, where "Never" corresponded to a score of 1, and "Always" represented a score of 5. The negatively worded questions were re-coded so that a higher score indicated higher practice. Nurses’ practices were assessed on a scale of 0 to 72, with higher scores indicating higher practice. The classification for nurses’ practice was defined as good (scores between 57.6 and 72, representing 80–100%) and poor (scores below 57.6, corresponding to ≤79%). Physicians’ practices were evaluated on a scale of 0 to 44, with good practice classified as scores between 35.2 and 44 (80–100%), and poor practice categorized as scores below 35.2 (≤79%) [29].

### Validation

For content validation, the questionnaire was distributed to 9 experts from various fields, including clinical pharmacy, community medicine, pharmacy practice, internal medicine, and an oncologist. The experts were asked to assess the relevance and representation of the items within their respective domains. The Scale-Content Validity Index based on the Universal Agreement method (S-CVI/UA) was employed for the knowledge and practices of physicians and nurses’ domains. The results of the S-CVI/UA were 0.78, 0.88, 0.87, and 0.81, respectively, and the Scale-Content Validity Index Average (S-CVI/Ave) were 0.96, 0.98, 0.97, and 0.97, respectively. This indicates a satisfactory level of content validity for the domains [30].

For face validation, three physicians and four nurses assessed the clarity and comprehension of the questions in each domain. Then, the questionnaire was piloted on ten physicians and ten nurses to evaluate its reliability. Cronbach’s alpha for the physicians’ knowledge and practices was calculated as 0.83 and 0.76, respectively. The calculated Cronbach’s alpha for the nurses’ knowledge and practices were 0.78 and 0.77, respectively, indicating that the questionnaire’ subscales had acceptable internal consistency. Feedback from pilot study participants and face validation helped to improve data collection for the main study.

### Procedure of data collection

The questionnaires were sent to the contact persons in all different areas of study with a cover letter written by the primary investigator describing the objectives of the study, and the name of the contact person. The questionnaires were distributed by the principal investigator and a well-trained data collectors’ team, who received adequate training on issues concerning the questionnaire (on the study’s objective, approaching the participants, and how to administer and collect the questionnaire timely). The questionnaires were handed to physicians and nurses in different centers and units after explaining the purpose of the study, and they were given a brief explanation about the study and its purpose after obtaining their verbal informed consent. Physicians and nurses were asked to complete the questionnaire and return it immediately after responding to it. Scoring was performed according to the number of correct answers.

### Statistical analysis

The data collected was analyzed statistically using descriptive statistics, namely mean with standard deviation for continuous variables, while the frequency with percentage for categorical variables. Content validity for all questionnaires was conducted using CVI-UA calculation. Cronbach’s alpha test ≥0.7 was used to check the reliability of the study questionnaire instruments and the consistency of the sample’s responses. A scoring system was implemented, wherein a pre-determined set of correct answers was used to assign scores to each category, allowing for quantitative assessment of knowledge and practice. The associations between the physicians’ and nurses’ overall knowledge and practice with their demographic data were analyzed using logistic regression models. Initially, univariate logistic regression was performed, and variables with a significance level (*P*-value < 0.25) were selected for inclusion in the multivariate logistic regression analysis. Odds ratios were calculated to determine the effect of each predictor on participants’ knowledge and practice. Correlation coefficients were calculated to assess the strength and direction of the relationship between physicians’ and nurses’ knowledge and practice using bivariate correlations, where strong: |r| ≥ 0.8, moderate: 0.5 < |r| < 0.8, and weak: |r| ≤ 0.5 [31]. A p-value less than 0.05 was used to indicate statistical significance. All statistical analyses were carried out using SPSS Version 25.0 (IBM Corp., Armonk, NY, USA), a widely used software for statistical analysis, see S3 and S4 Files.

## Results

### Demographic characteristics of nurses

A total of 105 nurses completed the questionnaire, giving a final response rate of 70%. Table 1 displays the demographic characteristics of the participants. The majority of nurses were male (61.0%), single (54.3%), had 1 to 5 years of experience (59.0%), and held a diploma degree (62.9%). Almost half of them (49.5%) fell within the 20–30 age range, and 34.3% worked in the daily administration ward. Moreover, slightly more than half of the participants (52.4%) had received chemotherapy training in the past five years. Among those, a significant proportion (61.9%) were unsure how many programs they had attended, while only 2.9% reported receiving more than five chemotherapy training programs. Furthermore, the majority of participants (70.5%) believed that the chemotherapy-related information received during their study was insufficient.

**Table 1:** Demographics characteristics of participants.

Nurse (n = 105)				Physicians (n = 77)			
Parameter		Frequency (n)	Percent (%)	Parameter		Frequency (n)	Percent (%)
Age				Age			
	20–30	52	49.5		25–35	14	18.2
	31–40	40	38.1		36–50	48	62.3
	More than 40	13	12.4		51–60	15	19.5
Sex					More than 60	0	0
	Male	64	61.0	Sex			
	Female	41	39.0		Male	54	70.1
Marital status					Female	23	29.9
	Married	42	40.0	Marital status			
	Single	57	54.3		Married	43	55.8
	Divorced	6	5.7		Single	30	39.0
Year of experience					Divorced	4	5.2
	1–5	62	59.0	Physicians’ category			
	6–9	20	19.0		Consultant	23	29.9
	10 or above	23	22		Specialist	34	44.1
Professional qualification					Resident	15	19.5
	Diploma	66	62.8		GP	5	6.5
	BSc	34	32.4	Physicians’ subspecialty			
	MSc	5	4.8		Medical Oncology	15	19.5
Working ward					Radiation Oncology	16	20.8
	Daily Administration	36	34.3		Surgical Oncology	14	18.2
	Clinic and Radiation	13	12.4		Pediatric oncology	9	11.7
	Admission	30	28.6		Hematology	9	11.7
	Preparation	24	22.8		Resident oncology	13	16.8
	ER	2	1.9		Specialist Assistance	1	1.3
In the past 5 years, have you received any chemotherapy training program?				Year of experience			
	Yes	55	52.4		Less than 3 Years	12	15.6
	No	50	47.6		3–5 Years	12	15.6
How many programs					6–10 Years	25	32.4
	1–2	28	26.7		More than 10 Years	28	36.4
	3–5	9	8.6	What is your main practice location where you spend most of your time?			
	More than 5	3	2.8		Office practice	3	3.9
	Not sure	65	61.9		Government Hospital	11	14.3
Do you think that information you have received about chemotherapy during your study was:					Oncology center	55	71.4
	Intensive	3	2.8		Private hospital/clinic	6	7.8
	Enough	15	14.3		Others	2	2.6
	Not enough	74	70.5				
	Not sure	13	12.4				

GP: general physician; ER: emergency room, n = frequency

### Demographic characteristics of physicians

The questionnaire was completed by 77 physicians, resulting in a final response rate of 89.53%. Table 1 displays the demographic characteristics of the participants. The majority of physicians were male (70.1%), married (55.8%), in the age range of 36–50 (62.3%), and worked in an oncology center (71.4%). Physicians with more than ten years of experience constituted the largest group (36.4%), followed by those with 6–10 years of experience (32.5%). The respondents were mainly specialists (44.2%), followed by consultants (29.9%), while radiation and medical oncology were the most common physicians’ subspecialties, with frequencies of 20.8% and 19.5%, respectively.

### Nurses’ knowledge about pharmacotherapy of Non-Hodgkin’s lymphoma

The overall knowledge level of nurses was assessed based on the proportion of correct answers. The results revealed that more than half of nurses (54.3%) demonstrated poor knowledge regarding NHL pharmacotherapy. In terms of the prevalence of NHL compared to HL, 40.0% of nurses incorrectly responded, and almost half of them (49.5%) provided incorrect responses about the hereditary nature of NHL. On the other hand, the majority of nurses (87.6% and 90.5%) correctly responded to the questions “Non-Hodgkin’s lymphoma is not contagious” and “Anti-emetic drugs should be administered 30 to 60min before NHL chemotherapy”, respectively (Table 2).

**Table 2:** Responses of nurses and physicians to the knowledge questions about pharmacotherapy of NHL.

Nurse Responses (n = 105)				Physicians’ Response (n = 77)			
Statement		Frequency	Percent%	Statement		Frequency	Percent%
Non-Hodgkin’s lymphoma (NHL) is not contagious.				Non-Hodgkin’s lymphoma (NHL) has more extranodal involvement compared to Hodgkin lymphoma (HL).			
	Incorrect answer	13	12.4		Incorrect answer	10	13.0
	Correct answer	92	87.6		Correct answer	67	87.0
NHL is more common than Hodgkin lymphoma (HL).				The majority of NHL patients present with peripheral lymphadenopathy.			
	Incorrect answer	42	40.0		Incorrect answer	32	41.6
	Correct answer	63	60.0		Correct answer	45	58.4
NHL is a hereditary disease (passed on directly from parents to children).				Immunocompromised patients are at increased risk of NHL.			
	Incorrect answer	52	49.5		Incorrect answer	13	16.9
	Correct answer	53	50.5		Correct answer	64	83.1
NHL can develop at all age periods: children, adults, and the elderly.				Diffuse large B-cell lymphoma (DLBCL) is the most common aggressive type of NHL.			
	Incorrect answer	21	20.0		Incorrect answer	20	26.0
	Correct answer	84	80.0		Correct answer	57	74.0
NHL stages are determined based on the number and location of lymphomas in the body.				The oral lesion is considered the main primary diagnostic factor of non-Hodgkin’s lymphoma.			
	Incorrect answer	30	28.6		Incorrect answer	22	28.6
	Correct answer	75	71.4		Correct answer	55	71.4
NHL most commonly appears as a solid tumor in the lymph nodes and can spread to extranodal tissues.				The goal of treatment for patients diagnosed with aggressive NHL is to cure.			
	Incorrect answer	20	19.0		Incorrect answer	35	45.5
	Correct answer	85	81.0		Correct answer	42	54.5
The most common symptom of NHL is a painless swelling in the neck, armpit, or groin.				The effective chemotherapy regimen for aggressive NHL is often complex, involving more than one agent.			
	Incorrect answer	35	33.3		Incorrect answer	8	10.4
	Correct answer	70	66.7		Correct answer	69	89.6
Chemotherapy is the mainstay of treatment in patients with NHL.				Radiation therapy has a limited role in NHL relative to HL since NHL is more often a systemic disease.			
	Incorrect answer	30	28.6		Incorrect answer	19	24.7
	Correct answer	75	71.4		Correct answer	58	75.3
Most often, the treatment of NHL is a regimen of 4 to 5 drugs known as CHOP/R-CHOP.				Patients with NHL of the central nervous system have a poor response to chemotherapy compared to those with other forms of NHL.			
	Incorrect answer	34	32.4		Incorrect answer	11	14.3
	Correct answer	71	67.6		Correct answer	66	85.7
NHL chemotherapy protocol contains oral and parenteral drugs.				Dosage adjustment for NHL patients based on body surface area and organ dysfunction (renal/hepatic impairment).			
	Incorrect answer	22	21.0		Incorrect answer	4	5.2
	Correct answer	83	79.0		Correct answer	73	94.8
Radiation therapy has a limited role in the treatment of NHL relative to HL.				The rituximab-containing regimen may reactivate latent diseases, such as hepatitis B.			
	Incorrect answer	37	35.2		Incorrect answer	27	35.1
	Correct answer	68	64.8		Correct answer	50	64.9
NHL is considered to be treatable.				Hyper-CVAD and EPOCH regimens are recommended for patients with HIV-related lymphoma.			
	Incorrect answer	19	18.1		Incorrect answer	61	79.2
	Correct answer	86	81.9		Correct answer	16	20.8
Mothers should not breastfeed while receiving NHL chemotherapy				R-CHOP is the first-line therapy for DLBCL with bulky and non-bulky stages.			
	Incorrect answer	28	26.7		Incorrect answer	10	13.0
	Correct answer	77	73.3		Correct answer	67	87.0
Dexamethasone drug can be used for NHL patients to prevent chemotherapy-induced nausea and vomiting.				ESHAP and DHAP are the more commonly salvage regimens used in patients with relapsed or refractory NHL.			
	Incorrect answer	31	29.5		Incorrect answer	15	19.5
	Correct answer	74	70.5		Correct answer	62	80.5
Anti-emetic drugs should be administered 30 to 60min before NHL chemotherapy.				Dexamethasone can be used to prevent acute and delayed CINV for moderately and highly emetogenic chemotherapy.			
	Incorrect answer	10	9.5		Incorrect answer	46	59.7
	Correct answer	95	90.5		Correct answer	31	40.3
				Mesna should be used to prevent cyclophosphamide-induced hemorrhagic cystitis.			
					Incorrect answer	28	36.4
					Correct answer	49	63.6
				Tumor lysis syndrome should be assessed during NHL treatment to prevent AKI.			
					Incorrect answer	5	6.5
					Correct answer	72	93.5
				Bleomycin-containing regimens may induce pulmonary fibrosis in NHL patients.			
					Incorrect answer	10	13.0
					Correct answer	67	87.0

NHL: Non-Hodgkin’s lymphoma, CINV: Chemotherapy-Induced Nausea and Vomiting, hyper-CVAD: hyper-fractionated cyclophosphamide, vincristine, doxorubicin, and dexamethasone, EPOCH: etoposide, prednisone, vincristine, cyclophosphamide, and doxorubicin, ESHAP: etoposide, methylprednisolone, high-dose cytarabine, and cisplatin, DHAP: dexamethasone, cisplatin, and cytarabine, AKI: Acute Kidney Injury.

### Physicians’ knowledge about pharmacotherapy of Non-Hodgkin’s lymphoma

The results of the study showed that physicians demonstrated a high level of knowledge regarding the question of dosage adjustment for NHL patients and the question of assessment of tumor lysis syndrome (TLS) during NHL treatment to prevent acute kidney injury (AKI), showing the highest correct response rates of 94.8% and 93.5%, respectively. However, a considerable percentage of physicians provided incorrect responses to the questions related to the presentation of NHL patients with peripheral lymphadenopathy and the treatment goal for patients diagnosed with aggressive NHL (41.6% and 45.5%), respectively. Surprisingly, the majority of physicians (79.2%) provided incorrect responses to the questions related to the recommended regimens for patients with HIV-related lymphoma and the use of dexamethasone to prevent acute and delayed chemotherapy-induced nausea and vomiting (59.7%), suggesting a knowledge gap in this area (Table 2). Overall, the analysis of physicians’ responses to knowledge questions revealed that 66.2% of physicians demonstrated poor knowledge regarding NHL pharmacotherapy.

### Nurses’ practice about pharmacotherapy of Non-Hodgkin’s lymphoma

The results indicated the following findings regarding nurses’ practice of pharmacotherapy of NHL. In terms of overall practice, the majority of nurses (83.8%) were categorized as having poor practice. Surprisingly, the results showed that more than half of nurses (52.4%) stated that they always practice chemotherapy administration before receiving proper training, and 41.9% of them always suggest exclusive treatment with alternative medicine for NHL patients. Moreover, 53.3% of nurses reported that they always substitute supportive care medications based on their availability, and a significant proportion of them (62.9%) never change to the correct way when they encounter medical errors while administering chemotherapy. Additionally, the results showed that the majority of participants (80%) always washed their hands and skin thoroughly after any contact with NHL chemotherapy (Table 3).

**Table 3:** Response of nurses and physicians to the practice questions about pharmacotherapy of NHL.

Nurses’ Responses (n = 105)				Physicians’ Response (n = 77)			
Statement		Frequency	Percent%	Statement		Frequency	Percent%
I do practice administering chemotherapy for NHL patients even before I receive the proper training.				How often do you use updated guidelines for treating NHL patients?			
	Always	43	41.0		Always	39	50.6
	Often	12	11.4		Often	13	16.9
	Sometimes	18	17.1		Sometimes	19	24.7
	Rarely	7	6.7		Rarely	2	2.6
	Never	25	23.8		Never	4	5.2
I do practice chemotherapy administration for NHL patients only based on my good experience.				Before prescribing chemotherapy medications for NHL patients, how often do you consider the potential drug-drug interactions and contraindications?			
	Always	56	53.3		Always	16	20.8
	Often	17	16.2		Often	47	61.0
	Sometimes	14	13.3		Sometimes	7	9.1
	Rarely	5	4.8		Rarely	3	3.9
	Never	13	12.4		Never	4	5.2
When I realize that a patient has NHL, I refer him/her directly to an oncologist.				How often do you prescribe off-label and/or unlicensed drugs for NHL patients?			
	Always	79	75.2		Always	6	7.8
	Often	11	10.5		Often	2	2.6
	Sometimes	5	4.8		Sometimes	12	15.6
	Rarely	1	1.0		Rarely	4	5.2
	Never	9	8.6		Never	53	68.8
When I realize that a patient has NHL, I suggest him/her to be treated exclusively with alternative medicine.				In your practice, how often do you change the chemotherapy protocol based on the desire of NHL patients?			
	Always	44	41.9		Always	3	3.9
	Often	2	1.9		Often	2	2.6
	Sometimes	6	5.7		Sometimes	27	35.1
	Rarely	9	8.6		Rarely	12	15.6
	Never	44	41.9		Never	33	42.9
I don’t prefer dealing with NHL patients who are experiencing serious side effects from chemotherapy.				In your practice, how often do you encounter that you are unable to order appropriate tests or treatments for NHL patients because of their cost?			
	Always	32	30.5		Always	7	9.1
	Often	6	5.7		Often	11	14.3
	Sometimes	16	15.2		Sometimes	37	48.1
	Rarely	18	17.1		Rarely	6	7.8
	Never	33	31.4		Never	16	20.8
Before the chemotherapy administration, I make sure that the NHL patient has used his/her pre-chemotherapy medications.				In your prescription, how often have you substituted chemotherapy medications for NHL patients due to their unavailability?			
	Always	71	67.6		Always	11	14.3
	Often	13	12.4		Often	4	5.2
	Sometimes	4	3.8		Sometimes	7	9.1
	Rarely	5	4.8		Rarely	24	31.2
	Never	12	11.4		Never	31	40.3
I prepare chemotherapy for NHL patients in a safe and appropriate area.				How often do you practice counseling for your NHL patients?			
	Always	81	77.1		Always	24	31.2
	Often	9	8.6		Often	26	33.8
	Sometimes	3	2.9		Sometimes	15	19.5
	Rarely	0	0		Rarely	3	3.9
	Never	12	11.4		Never	9	11.7
I do not eat, drink, or smoke in areas where NHL chemotherapy is administered.				How often do you provide nurses and/or other health care practitioners with a comprehensive summary, including detailed information about NHL treatment?			
	Always	76	72.4		Always	23	29.9
	Often	6	5.7		Often	9	11.7
	Sometimes	9	8.6		Sometimes	31	40.3
	Rarely	3	2.9		Rarely	9	11.7
	Never	11	10.5		Never	5	6.5
I wash my hands and skin thoroughly after any contact with NHL chemotherapy.				How often do you closely supervise nurses and/or other health care practitioners while they are administering chemotherapy to patients with NHL to ensure proper and correct administration?			
	Always	84	80.0		Always	19	24.7
	Often	7	6.7		Often	11	14.3
	Sometimes	3	2.9		Sometimes	34	44.2
	Rarely	1	1.0		Rarely	4	5.2
	Never	10	9.5		Never	9	11.7
When I administer NHL chemotherapy, I wear personal protective tools, such as gloves & mask.				How often do you follow up directly with NHL patients while they are receiving chemotherapy to avoid any adverse effects or complications that can occur for patients?			
	Always	79	75.2		Always	40	51.9
	Often	10	9.5		Often	16	20.8
	Sometimes	7	6.7		Sometimes	14	18.2
	Rarely	1	1.0		Rarely	2	2.6
	Never	8	7.6		Never	5	6.5
I receive direct instructions and follow-ups from oncologists while administering chemotherapy to NHL patients.				How often do you perform surveillance and screening tests for an NHL survivor who is currently asymptomatic?			
	Always	65	61.9		Every 6 months	31	40.3
	Often	8	7.6		Yearly	24	31.2
	Sometimes	21	20.0		Only If indicated	7	9.1
	Rarely	5	4.8		Never	4	5.2
	Never	6	5.7		Don’t Know	4	5.2
	-	-	-		Other	7	9.1
During administration, I change some of the supportive care medications prescribed for NHL patients according to what is available at the NOC.				What type of medical records system do you use during your practice?			
	Always	56	53.3		Paper records and charts	42	54.5
	Often	13	12.4		Partial electronic medical records	10	13.0
	Sometimes	26	24.8		In the transition from paper to full electronic medical records	23	29.9
	Rarely	7	6.7		Full electronic medical records	2	2.6
	Never	3	2.9		-	-	-
I monitor NHL patients regularly during their chemotherapy administration.				In the past 5 years, have you participated in training activities regarding NHL therapy?			
	Always	74	70.5		Yes	63	81.8
	Often	12	11.4		No	14	18.2
	Sometimes	5	4.8	In which of the following training activities have you participated? Medical conferences			
	Rarely	1	1.0		Yes	30	39.0
	Never	13	12.4		No	47	61.0
I support NHL patients psychologically and socially during their chemotherapy administration.				Medical Research			
	Always	82	78.1		Yes	8	10.4
	Often	12	11.4		No	69	89.6
	Sometimes	4	3.8	Postgraduate medical training			
	Rarely	2	1.9		Yes	10	13.0
	Never	5	4.8		No	67	87.0
I deal with chemotherapy complications for NHL patients professionally				Scientific meetings			
	Always	59	56.2		Yes	39	50.6
	Often	19	18.1		No	38	49.4
	Sometimes	11	10.5	Medical Websites and Programs			
	Rarely	1	1.0		Yes	5	6.5
	Never	15	14.3		No	72	93.5
When I recognize a medical error during chemotherapy administration, I keep administering medication anyway.				Are you currently involved with teaching medical students and/or residents?			
	Always	78	74.3		Yes	46	59.7
	Often	4	3.8		No	31	40.3
	Rarely	0	0	What is the main problem you encounter during your practice that might significantly affect the treatment outcomes of NHL patients?			
	Rarely	1	1.0	Side effects of chemotherapy			
	Never	22	21.0		Yes	41	53.2
When I recognize a medical error during chemotherapy administration, I contact the physician.					No	36	46.8
	Always	72	68.6	Patient’s noncompliance/ignorance			
	Often	9	8.6		Yes	29	37.7
	Sometimes	6	5.7		No	48	62.3
	Rarely	0	0	Unreliable diagnostic test results			
	Never	18	17.1		Yes	7	9.1
When I recognize a medical error during chemotherapy administration, I change it in the right way.					No	70	90.9
	Always	25	23.8	Inappropriate administration of chemotherapy			
	Often	4	3.8		Yes	6	7.8
	Sometimes	8	7.6		No	71	92.2
	Rarely	2	1.9	Unavailability of chemotherapy medications			
	Never	66	62.9		Yes	27	35.1
					No	50	64.9
				High cost of medications and diagnostic lab tests			
					Yes	19	24.7
					No	58	75.3
				The psychological and economic condition of the patients			
					Yes	19	24.7
					No	58	75.3
				Others:			
				In-availability of some diagnostic tools PET-CT		10	13.0
				None		5	6.5

NHL: Non-Hodgkin’s lymphoma, PET-CT: Positron Emission Tomography-Computed Tomography

### Physicians’ practice about pharmacotherapy of Non-Hodgkin’s lymphoma

In terms of overall practice, only 24.7% of physicians demonstrated good practice, while 75.3% exhibited poor practice regarding pharmacotherapy of NHL. The majority of physicians (81.8%) have participated in different training activities related to NHL pharmacotherapy within the past five years. Among these activities, scientific meetings and medical conferences were reported by (50.6% and 39.0%) of physicians, respectively. However, participation in postgraduate medical research and medical training was only reported by (10.4% and 13.0%) of the physicians, respectively. In addition, a significant proportion of physicians (54.5%) reported that they use paper records and charts as medical record systems. The most common challenges reported by physicians that could significantly affect NHL treatment outcomes are chemotherapy side effects (53.2%), patients’ non-compliance/ignorance (37.7%), and unavailability of chemotherapy medications (35.1%) (Table 3).

### Factors associated with nurses’ knowledge and practice

Logistic regression analysis was conducted to examine the relationship between various variables and the likelihood of having good knowledge or practice among nurses in pharmacotherapy for NHL (Table 4). For nurses, both univariate and multivariate logistic regression analyses were performed. The results of the univariate model indicated that nurses who had more than nine years of experience and received enough information about chemotherapy during their study were more likely to have good knowledge compared to the reference group (p = 0.012, OD = 3.571, CI = 1.323–9.639) and (p = 0.002, OD = 8.182, CI = 2.201–30.416), respectively. After adjusting for other factors in the multivariate model, the analysis revealed that only one variable, receiving sufficient information about chemotherapy during their study, remained significantly and independently associated with total knowledge (p = 0.037, AOD = 5.170, CI = 1.105–24.203).

**Table 4:** Binary regression for total knowledge and practice of nurses.

Variable		Total knowledge		Univariate logistic regression		Multivariate logistic regression		Total practice		Univariate logistic regression		Multivariate logistic regression	
				OR (95% CI)	p. value	AOR (95% CI)	p. value			OR (95% CI)	p. value	AOR (95% CI)	p. value
		Poor knowledge %	Good knowledge%					Poor practice %	Good practice%				
Age													
	≤ 30	33 (63.5%)	19 (36.5%)	Reference		Reference		43 (82.7%)	9 (17.3%)	Reference			
	> 30	24 (45.3%)	29 (54.7%)	2.099 (.960–4.586)	0.063	1.148 (.420–3.142)	.788	45 (84.9%)	8 (15.1%)	.849 (.300–2.403)	.758		
Sex													
	Male	32 (50.0%)	32 (50.0%)	Reference		-	-	54 (84.4%)	10 (15.6%)	Reference			
	Female	25 (61.0%)	16 (39.0%)	.640 (.289–1.419)	.272	-	-	34 (82.9%)	7 (17.1%)	1.112 (.386–3.199)	.844		
Marital status													
	Single	32 (56.1%)	25 (43.9%)	Reference		-	-	46 (80.7%)	11 (19.3%)	Reference			
	Married/Divorced	25 (53.2%)	22 (46.8%)	1.126 (.518–2.447)	.764	-	-	41 (87.2%)	6 (12.8%)	.612 (.208–1.802)	.373		
Year of experience													
	≤ 9	50 (61.0%)	32 (39.0%)	Reference				71 (86.6%)	11 (13.4%)	Reference		Reference	
	>9	7 (30.4%)	16 (69.6%)	3.571 (1.323–9.639)	.**012**	1.772 (.482–6.510)	.389	17 (73.9%)	6 (26.1%)	2.278 (.738–7.029))	.152	2.051 (.642–6.556)	.226
Professional qualification													
	Diploma	39 (59.1%)	27 (40.9%)	Reference		Reference		56 (84.8%)	10 (15.2%)	Reference			.
	BSc/MSc	18 (46.2%)	21 (53.8%)	1.685 (.759–3.744)	.200	.907 (.344–2.392)	.844	32 (82.1%)	7 (17.9%)	1.225 (.425–3.532)	.707		
Working ward													
	Others wards	42 (60.9%)	27 (39.1%)	Reference				62 (89.9%)	7 (10.1%)	Reference		Reference	
	Daily Administration	15 (41.7%)	21 (58.3%)	2.178 (.959–4.946)	.063	1.869 (744–4.698)	.183	26 (72.2%)	10 (27.8%)	3.407 (1.170–9.921)	**.025**	3.234 (1.099–9.515)	**.033**
Governorate of center/unit													
	Other Cities	42 (60.0%)	28 (40.0%)	Reference		Reference		58 (82.9%)	12 (17.1%)	Reference			
	Sana’a-NOC	15 (42.9%)	20 (57.1%)	2.000 (.878–4.553)	.099	1.980 (.741–5.293)	.173	30 (85.7%)	5 (14.3%)	.806 (.260–2.500)	.708		
Training programs													
	No	31 (62.0%)	19 (38.0%)	Reference		Reference		41 (82.0%)	9 (18.0%)	Reference			
	Yes	26 (47.3%)	29 (52.7%)	.549 (.252–1.197)	.132	.638 (.199–2.042)	.449	47 (85.5%)	8 (14.5%)	.775 (.274–2.195)	.632		
No. of Training programs													
	≤ 2	11 (39.3%)	17 (60.7%)	Reference		Reference		25 (89.3%)	3 (10.7%)	Reference			
	>2	46 (59.7%)	31 (40.3%)	.436 (.180–1.056)	.066	.368 (.101–1.341)	.130	63 (81.8%)	14 (18.2%)	1.852 (.490–7.004)	.364		
Information received:													
	Not enough	54 (62.1%)	33 (37.9%)	Reference		Reference		74 (85.1%)	13 (14.9%)	Reference			
	Enough	3 (16.7%)	15 (83.3%)	8.182 (2.201–30.416)	**.002**	5.170 (1.105–24.203)	**.037**	14 (77.8%)	4 (22.2%)	.615 (.175–2.163)	.449		

Variables of <0.25 in the univariate analysis were included in the multivariable regression model. Method used for regression: (Enter), OR (Odds Ratio), CI (Confidence Interval); p-value in bold means it is significant.

Regarding total practice among nurses, the univariate analysis model showed that nurses working in the daily administration ward were more likely to have good practice compared to the reference group (p = 0.025, OR = 3.407, CI = 1.170–9.921). Subsequently, multivariate regression analysis was performed to identify the variables significantly and independently associated with practice. The multivariate regression analysis revealed that only working in the daily administration ward fulfilled the criteria, indicating a significant association with good practice (p = 0.033, AOD = 3.234, CI = 1.099–9.515).

### Factors associated with physicians’ knowledge and practice

The study indicated the following findings regarding factors associated with physicians’ knowledge about pharmacotherapy for NHL (Table 5). In univariate analysis, the results showed that physicians who were single/divorced were more likely to have good knowledge compared to the reference group (p = 0.031, OD = 2.933, CI = 1.104–7.790). After adjusting for other factors in the multivariate model, the results revealed that being single/divorced was the only variable significantly and independently associated with total knowledge among physicians (p = 0.041, AOD = 2.838, CI = 1.041–7.735). Furthermore, the univariate analysis for physicians demonstrated that physicians over 50 years old and those working in the NOC in Sana’a were more likely to have good practice compared to the reference group (p = 0.034, OD = 3.646, CI = 1.105–12.034) and (p = 0.004, OD = 20.667, CI = 2.585–165.214), respectively. Surprisingly, consultants were less likely to have good practice than specialists, residents, and GP group (p = 0.016, OD = 3.846, CI = 1.291–11.459). After adjusting for other factors in the multivariate model, the multivariate regression analysis identified the working in the NOC in Sana’a as the only variable significantly and independently associated with total practice among physicians (p = 0.009, AOD = 21.961, CI = 2.195–219.758).

**Table 5:** Binary logistic regression for total knowledge and practice of physicians.

Variable		Total knowledge		Univariate logistic regression		Multivariate logistic regression		Total practice		Univariate logistic regression		Multivariate logistic regression	
				OR (95% CI)	p. value	AOR (95% CI)	p. value			OR (95% CI)	p. value	AOR (95% CI)	p. value
		Poor knowledge %	Good knowledge%					Poor practice %	Good practice%				
Age													
	≤ 50	41 (66.1%)	21 (33.9%)	Reference				50 (80.6%)	12 (19.4%)	Reference			
	> 50	10 (66.7%)	5 (33.3%)	.976 (.295–3.226)	.968			8 (53.3%)	7 (46.7%)	3.646 (1.105–12.034)	**.034**	2.689 (.515–14.033)	.241
Sex													
	Male	38 (70.4%)	16 (29.6%)	Reference		Reference		40 (74.1%)	14 (25.9%)	Reference			
	Female	13 (56.5%)	10 (43.5%)	1.827 (.665–5.018)	0.242	1.606 (.521–4.950)	.410	18 (78.3%)	5 (21.7%)	0.79(0.24–2.53)	0.697		
Marital status													
	Married	33 (76.7%)	10 (23.3%)	Reference				29 (67.4%)	14 (32.6%)	Reference			
	Single /Divorced	18 (52.9%)	16 (47.1%)	2.933 (1.104–7.790)	**.031**	2.838 (1.041–7.735)	**.041**	29 (85.3%)	5 (14.7%)	.357 (.114–1.121)	.078	.388 (.100–1.513)	.173
Year of experience													
	≤ 5	17 (70.8%)	7 (29.2%)	Reference				21 (87.5%)	3 (12.5%)	Reference		Reference	
	> 5	34 (64.2%)	19 (35.8%)	1.357 (.478–3.855)	.566			37 (69.8%)	16 (30.2%)	3.027 (.789–11.611)	.106	.570 (.097–3.338)	.533
Physicians’ category													
	Others	17 (73.9%)	6 (26.1%)	Reference				13 (56.5%)	10 (43.5%)	Reference			
	Consultant	34 (63.0%)	20 (37.0%)	.600 (.203–1.771)	.355			45 (83.3%)	9 (16.7%)	3.846 (1.291–11.459)	**.016**	.622 (.140–2.761)	.532
Physicians’ subspecialty													
	Others	40 (64.5%)	22 (35.5%)	Reference				46 (74.2%)	16 (25.8%)	Reference			
	Medical Oncology	11 (73.3%)	4 (26.7%)	.661 (.188–2.324)	.519			12 (80.0%)	3 (20.0%)	.719 (.180–2.877)	.641		
Governorate of center/unit													
	Other Cities	18 (56.3%)	14 (43.8%)	Reference		Reference		31 (96.9%)	1 (3.1%)	Reference		Reference	
	Sana’a-NOC	33 (73.3%)	12 (26.7%)	.468 (.179–1.223)	0.121	.616 (.213–1.784)	.372	27 (60.0%)	18 (40.0%)	20.667 (2.585–165.214)	**0.004**	21.961 (2.195–219.758)	**.009**
Main practice location													
	Outside Oncology Center	16 (72.7%)	6 (27.3%)	Reference				17 (77.3%)	5 (22.7%)	Reference			
	In Oncology Center	35 (63.6%)	20 (36.4%)	1.524 (.514–4.520)	0.448			41 (74.5%)	14 (25.5%)	1.161 (.361–3.730)	.802		

Variables of <0.25 in the univariate analysis were included in the multivariable regression model. Method used for regression: (Enter), OR (Odds Ratio), CI (Confidence Interval); p-value in bold means it is significant.

### Correlation between nurses’ and physicians’ knowledge and practice

There is a weak significant correlation between nurses’ knowledge and practice (r = 0.343, P-value < 0.001). The correlation coefficient (r) is reported as 0.343, indicating a positive correlation between nurses’ knowledge and their practice. This means that as nurses’ knowledge increases, their practice also tends to improve (Table 4). Based on the results among physicians, there is no significant correlation between physicians’ knowledge and practice (r = 0.205, P-value = .074) (Table 6).

**Table 6:** Correlation between nurses’ and physicians’ knowledge and practice.

Variable	Nurses (n = 105)	Physicians (n = 77)
	Practice	Practice
Knowledge	r ^a^:0.343*	r ^a^:0.205
	P value: <0.001	P value: 0.074

^a^ Spearman correlation

* indicates significant correlation.

## Discussion

The results of this study described the knowledge and practice of nurses and physicians regarding the pharmacotherapy of NHL as a multi-center study in eight centers and units in Yemen. Almost half of the nurses have not received chemotherapy training courses within the past five years. This insufficient training often raises concerns about their abilities and skills in administering chemotherapy to cancer patients, as chemotherapy administration is a complex and risky process due to expected high levels of adverse effects, resulting in errors and suboptimal treatment outcomes. This requires continuous training and awareness-raising among healthcare professionals, especially nurses [32]. Possible reasons for this lack of training may include limited access to training programs, limitations of available resources, and staff shortages [20]. These findings support a previous study in Bangladesh, which found that most nurses in Bangladesh lack access to chemotherapy training programs. Similarly, another study conducted in Poland highlighted that employers do not provide funding for training nurses for NHL, which may lead to insufficient knowledge and skills in patient care [33, 34].

### Nurses’ knowledge about pharmacotherapy of NHL

The study proved that the majority of nurses do not have sufficient knowledge regarding the pharmacotherapy of NHL. Such a gap in their knowledge and experience in this field raises doubts about their competence and ability to practice chemotherapy administration effectively. These results contrast with a previous study conducted in Taiz, Yemen, which revealed that half of the nurses had good knowledge regarding the management of side effects of chemotherapy [35]. The possible explanations for this discrepancy in the knowledge of nurses across Yemen include differences in sample size, nurses’ backgrounds, study locations, and differences in education, training, and resources that may also influence these disparities. On the other hand, this finding is consistent with previous studies conducted in Poland and Pakistan that highlighted insufficient and poor knowledge about NHL among nurses [23, 33]. These consistent findings underscore the need for targeted education and training programs to improve nurses’ knowledge and awareness of NHL drug therapy.

Moreover, this study evaluated nurses’ understanding of the hereditary nature of NHL and found that 49.5% of nurses mistakenly believed that NHL is a hereditary disease passed directly from parents to children. However, it is important to clarify that NHL is a malignancy originating from immune cells and is not commonly considered hereditary. While a slightly increased risk is observed in family members, NHL is not classified as a hereditary disorder [36–38]. These findings emphasize the importance of enhancing nurses’ understanding of pharmacotherapy for NHL, similar to a previous study conducted in Poland that emphasized the necessity for education on NHL among nurses [33]. While nurse-led chemotherapy education is essential for optimal cancer care, current guidelines primarily focus on treatment specifics. Nurses play a vital role in addressing these concerns, answering questions, and providing support, as patients frequently seek guidance on adapting their daily routines for safety and improved well-being during chemotherapy [15]. In addition, oncology nurses with good knowledge of chemotherapy-induced peripheral neuropathy contribute to improving patients’ quality of life [39].

### Nurses’ practice about pharmacotherapy of Non-Hodgkin’s lymphoma

The study findings raise significant concerns about the practice of nurses in NHL pharmacotherapy. The majority of nurses showed poor practice, indicating the need to improve their knowledge and implement appropriate protocols of pharmacotherapy. This finding is in agreement with a previous study in Erbil, which revealed that the majority of participants demonstrated poor practice in the safe handling of chemotherapy [24]. It is alarming to note that a considerable percentage of nurses administer chemotherapy to patients without undergoing proper training. This situation poses potential risks to patient safety and emphasizes the critical need for nurses to receive adequate training before handling chemotherapy medications. Ensuring the appropriate level of nurses’ knowledge is essential for delivering proper nursing care and effective health education [33].

Another concern in the finding is that a significant proportion of nurses in the study recommended exclusive treatment with alternative medicine for NHL patients. This is consistent with previous studies by Damkier et al. and Fitch et al., which observed that nurses perceive the use of alternative medicine as a personal choice [40, 41]. Nurses’ recommendation of exclusive alternative medicine for NHL patients may deviate from evidence-based practice and compromise standard pharmacotherapy effectiveness. This highlights a lack of basic knowledge regarding alternative medicine among nurses, consistent with a previous study in Qatar emphasizing the need to enhance complementary therapy education for oncology nurses [42]. On the other hand, Canadian oncology nurses acknowledge the importance of complementary and alternative medicine as part of person-centered care, emphasizing its inclusion in the foundations of oncology nursing practice. These findings underscore the need for comprehensive education and integration of complementary therapies into the training and practice of oncology nurses [43]. In a previous study conducted in Erbil, concerns in practices were observed, including inadequate changing of protective barriers after chemotherapy contact and a lack of knowledge regarding chemotherapy contamination through food [24]. Similarly, a study in Bangladesh reported inadequate use of specially designed personal protective equipment when handling chemotherapy agents [34]. In contrast, our study demonstrated positive results. The majority of respondents consistently used personal protective tools, such as gloves and masks, when administering NHL chemotherapy. They also exhibited good hygiene practices by thoroughly washing their hands and skin after any contact with NHL chemotherapy. In Bangladesh, approximately two-thirds of respondents (66.7%) prepared chemotherapeutic agents in a safe environment. Similarly, in our study, most respondents (77.1%) reported always preparing chemotherapy for NHL patients in a safe and appropriate area [34]. Nurses handling chemotherapy drugs should have appropriate qualifications and training to enhance safety standards. To improve their knowledge, all oncology nurses are required to undergo nursing education. As nurses continuously gain more knowledge, they are more likely to follow safety protocols when dealing with the administration of cancer chemotherapy, which can lead to improved health outcomes [34].

Overall, these results suggest the need for targeted interventions, including further training, education, and the establishment of clear guidelines, to promote safe and effective pharmacotherapy practices among nurses in NHL treatment. Educational institutions and nursing programs should prioritize providing nurses with a robust understanding of chemotherapy through coursework, practical training, and access to up-to-date resources. Equipping nurses with early education and training enables them to provide effective, evidence-based care to patients [44].

### Physicians’ knowledge about pharmacotherapy of Non-Hodgkin’s lymphoma

The study findings demonstrated varying levels of knowledge among physicians regarding the pharmacotherapy of NHL in multi-centers in Yemen. The questions regarding dosage adjustment for NHL patients and the question of assessment of TLS during NHL treatment to prevent AKI showed the highest correct response rates. These findings indicate that the participants have a solid understanding of these critical aspects of NHL management. Such knowledge is crucial for delivering safe and effective care to patients with NHL. The high response rates to these questions indicate that participants have a strong understanding of two critical aspects of managing NHL: dose adjustment and prevention of TLS. Dosage adjustment ensures appropriate chemotherapy dosing for individual patients, considering factors like body surface area and organ dysfunction. TLS prevention involves monitoring for signs and implementing measures to mitigate TLS risks. Participants’ knowledge in these areas reflects their readiness to provide safe and effective care for NHL patients [45, 46].

However, physicians had a notable knowledge gap regarding the recommended regimens for treating NHL in patients with HIV-related lymphoma. Managing patients with HIV-related lymphoma poses a challenging situation, especially when many present with high-grade NHL. A typical manifestation involves extranodal disease, often affecting the gastrointestinal tract, central nervous system (CNS), or bone marrow [47]. Due to the aggressive nature of HIV-related lymphoma, it is recommended to employ intensive chemotherapy protocols such as Hyper-CVAD (hyper-fractionated cyclophosphamide, vincristine, doxorubicin, and dexamethasone) and EPOCH (etoposide, prednisone, vincristine, cyclophosphamide, and doxorubicin) [5]. Despite the higher prevalence of non-Hodgkin lymphoma in HIV-infected individuals compared to the general population [48], physicians’ limited exposure to HIV-related lymphoma cases and their lack of experience and specialized training or education specifically focused on HIV-related lymphoma may contribute to their insufficient understanding of recommended treatment regimens. Surprisingly, a significant proportion of physicians provided incorrect responses regarding the use of dexamethasone to prevent acute and delayed chemotherapy-induced nausea and vomiting (CINV). According to the recommendations issued by the National Comprehensive Cancer Network [45] and the American Society of Clinical Oncology [49, 50], dexamethasone is advised to be used for CINV prophylaxis in the HEC and MEC settings. The lack of awareness of current guidelines among physicians could explain these findings. Consistent with these findings, a previous study has shown that the guideline-recommended triplet of NK1RA–5-HT3RA-dexamethasone was prescribed in only 12.2% of cycles in five European countries: France, Germany, Italy, Spain, and the UK [47]. Continuing education in the healthcare field is emphasized as vital for healthcare workers to assume responsibility for their development, achieve competence individually and as a team, and enhance their skills and knowledge. Ultimately, this leads to an improved quality of healthcare delivery [51].

In terms of the overall knowledge assessment, the analysis of physicians’ knowledge regarding NHL pharmacotherapy indicated that a significant proportion of physicians showed good knowledge. Similarly, a study conducted in Bangladesh reported that 54.15% of the participants achieved a good or above-average score in the knowledge section [11].

### Physicians’ practice about pharmacotherapy of Non-Hodgkin’s lymphoma

The findings in this study indicated that only a small percentage of physicians exhibited good practice, while the majority (75.3%) showed poor practice regarding NHL pharmacotherapy. This emphasizes the need to improve the quality of care physicians provide in this area. In contrast, a study in Bangladesh found that 65.54% of participants scored above average, indicating that community healthcare providers and health assistants effectively fulfilled their roles with good practices [11]. Regarding training activities, the majority of physicians (81.8%) in this study reported participating in NHL therapy-related training activities within the past five years, primarily through medical conferences and scientific meetings. This demonstrates their commitment to ongoing education. However, over 90% of community healthcare providers and health assistants in Bangladesh did not receive cancer-related training from the government [11]. In this study, the majority of physicians (71.5%) provide regular follow-up of patients with NHL, even if they were asymptomatic, conducting surveillance and screening tests every six months or yearly. This is consistent with a similar study in Bangladesh, where a majority of community healthcare providers and health assistants (84.25% and 85.35%, respectively) conducted follow-ups for identified cancer patients during fieldwork [11]. However, contrasting results were observed in a study conducted in Lazio, Italy, where many physicians did not provide follow-up care for patients who tested positive for cancer [49]. Long-term follow-up care for cancer survivors is critical, as guidelines emphasize continuous follow-up for the first five years after treatment and less frequent follow-up after that [52, 53].

Overall, these results suggest that physicians need to improve their practice regarding NHL pharmacotherapy. This could be done through continuous education, addressing side effects and patient compliance, and ensuring medication availability. By improving their practice, physicians can improve treatment outcomes for patients.

### Factors associated with the knowledge and practice of nurse

The significant findings of the logistic regression analysis revealed the association between nurses’ receiving sufficient information during their education and their level of knowledge. This relationship remains statistically significant, as indicated by the significantly adjusted odds ratio, even after considering other factors. In the specific context of oncology nursing, previous research supported the importance of acquiring adequate information through continuous educational activities such as seminars and workshops. These activities play a crucial role in increasing knowledge and strengthening competencies, particularly in the safe administration of chemotherapy and adopting a proactive approach to managing side effects. By actively participating in continuous education, oncology nurses can enhance their skills and ultimately contribute to improved patient outcomes [16]. Moreover, the findings highlighted the complex interplay of various factors in nurses’ practice. Among these factors, years of experience and working ward emerged as influential, where nurses with more experience and those working in the daily administration wards demonstrated a higher percentage of good practice. Several explanations can be attributed to the finding that nurses in the daily administration ward exhibit a higher percentage of good practice than others. One possible reason is their regular involvement in administering chemotherapy to patients, as the daily administration ward in the oncology center is where most chemotherapy treatments are provided to patients, which enhances their understanding and implementation of best practices. Additionally, the work environment in the daily administration ward may also offer more support and resources for nurses to gain experience, enabling them to deliver high-quality care during administration. This underscores the importance of ensuring excellence in nursing practices within this department.

### Factors associated with the knowledge and practice of physicians

The logistic regression analysis revealed significant factors associated with physicians having good knowledge and good practice compared to the reference group. Regarding the marital status of physicians, single/divorced physicians showed higher knowledge than married physicians. Single/divorced physicians could have advantages regarding time availability for studies and research, higher motivation to succeed in their careers, and better access to educational resources [54]. However, they may have less good practice due to increased stress levels and long working hours, which can lead to fatigue and impact patient care. Addressing these challenges can help improve the practice of single/divorced physicians in oncology [54].

Furthermore, a previous study conducted in Eswatini noted that community health workers in Lubombo, the legislative capital, had slightly lower knowledge scores than their counterparts in Hhohho [55]. Similarly, our study focused on physicians working on the NOC in Sana’a, the capital of Yemen, and revealed a similar trend. It was found that these physicians are less likely to have good knowledge compared to physicians working in other governorates and different regions across Yemen. This discrepancy in levels of knowledge among physicians may be attributed to various environmental factors, including limited access to educational resources due to the ongoing conflict in Yemen, high staff attrition rates, and potential difficulties in obtaining professional development opportunities. However, despite this relative lack of knowledge, it is important to highlight that these physicians in the NOC appear to have more practical experience than their counterparts in other governorates. The possible explanation for that includes a greater opportunity to deal with various conditions, giving them more hands-on experience, as the NOC in Sana’a treats a higher number of cases than other hospitals.

### Correlation between nurses’ and physicians’ knowledge and practice

A correlation coefficient of 0.343 indicates a positive association between nurses’ knowledge and practice. As well-informed nurses, their practices improve accordingly, suggesting that staying up-to-date can positively impact patient care and outcomes, as the more knowledgeable nurses are, the more they use safety measures in their practice and better patient outcomes [23]. One possible explanation for this positive correlation is that nurses who strongly understand evidence-based medical concepts, procedures, and practices are more likely to apply their knowledge effectively in their daily clinical practice. By keeping abreast of the latest developments in their field, nurses can make informed decisions, provide optimal patient care, and contribute to positive patient outcomes. In contrast to our study, the result of a previous study conducted in Erbil city showed a significant negative association between knowledge and practices among oncology nurses (r = -0.469, p = 0.014) [24].

### Study limitations

First, the convenience sampling method and the small sample size may affect the generalizability of our findings. These limitations can be attributed to the difficulty in obtaining a larger and more representative sample due to the rarity of NHL in Yemen. Additional challenges during the study’s conduct included the complicated treatment regimen for NHL patients and the limited cooperation of some physicians. The study was only conducted in a specific geographic region, Yemen, which may have limited the application of the findings to other contexts and populations. Nevertheless, despite these limitations, our research provides significant relevance since it sheds light on important relationships between physicians’ clinical practice and knowledge of the pharmacotherapy of NHL. It establishes the foundation for ongoing research and serves as a starting point for larger, more varied future investigations intended to both confirm our findings and overcome these acknowledged limitations. Moreover, the multi-center design of the study strengthens the breadth of the data collected and contributes to a richer, more nuanced understanding of the current practices and knowledge gaps in NHL pharmacotherapy in Yemen. Also, the study’s focus on both physicians and nurses provides a more comprehensive view of healthcare providers’ knowledge and practice in this area.

## Conclusion

In conclusion, the study revealed alarming knowledge gaps and concerning practices in NHL pharmacotherapy among nurses and physicians in Yemen. While physicians demonstrated high knowledge in certain areas like dosage adjustment, a considerable portion displayed knowledge gaps related to treatment goals and recommended regimens. Nurses exhibited overall poor knowledge, with many lacking recent chemotherapy training and awareness about NHL pharmacotherapy. Targeted educational interventions and continuous training are essential to enhance clinicians’ and nurses’ knowledge and practices, thereby improving patient care.

## Supporting information

S1 File Nurses’ questionnaire. (DOCX)

S2 File Physicians’ questionnaire. (DOCX)

S3 File Nurses’ dataset. (XLSX)

S4 File Physicians’ dataset. (XLSX)
