# Supplementary material for: Evaluation of clinicians’ knowledge and practice regarding pharmacotherapy of Non-Hodgkin’s lymphoma: A multi-center study in Yemen
Source: PLoS One. 2024 Jun 5;19(6):e0304209. doi: 10.1371/journal.pone.0304209 (PMC11152296; doi:10.1371/journal.pone.0304209)
Supplement: S1 File — (DOCX) [file pone.0304209.s001.docx]

**Assessment the knowledge and practice of Nurses regarding pharmacotherapy of NHL**

This study aims to assess nurses’ knowledge and practice regarding NHL pharmacotherapy in Sana’a, Yemen.

| **Section 1: Demographic Data**  **البيانات الديموغرافية** | | |
| --- | --- | --- |
| **Age (Years): …………..**  **العمر بالسنوات** | **Gender:** 🞎 **Male** 🞎 **Female**  الجنس | Marital Status: 🞎 Single 🞎 Married 🞎 Divorced  الحالة الاجتماعية |
| **Years of Experience as a nurse in the oncology-related field:**  **سنوات الخبرة كممرض/كممرضة في مجال الأورام ……………………** | | |
| **Professional Qualification المؤهلات المهنية**  🞎 **Diploma** 🞎 **BSc** 🞎 **MSc** 🞎 **PhD** | | |
| **Nurse’s working ward: القسم الذي تعمل فيه**  🞎 **Daily administration** 🞎 **Clinics/Radiation** 🞎**Daily Admission wards** 🞎 **Preperation ward**  **أقسام الرقود العيادات /الاشعاع قسم الاعطاء اليومي قسم التحضير** | | |
| **In the past 5 years, have you received any chemotherapy training program?**  **هل تلقيت أي برنامج تدريبي في العلاج الكيماوي خلال الخمس السنوات الماضية؟**  🞎 **Yes** 🞎 **No** 🞎 **I’m not sure**  **If your answer (Yes), How many programs? (Please specify) …………………………………….**  **إذا كانت الإجابة نعم، كم عدد البرامج التي تلقيتها؟** | | |
| **Do you think that information you have received about chemotherapy during your study was:**  **هـل تعتقد أن المعلومات التي تلقيتها عن العلاج الكيماوي أثناء دراستك كانت:**  🞎 **مكثفة Intensive** 🞎 **كافية Enough** 🞎 **غير كافية Not-enough** 🞎 **لست متأكد I’m not sure** | | |

| **Section 2: Questions related to the assessment of nurses' knowledge regarding NHL pharmacotherapy** | | | | |
| --- | --- | --- | --- | --- |
| I don’t know  لا اعلم | Disagree  غير موافق | Agree  موافق | **Knowledge**  **المعرفـــــــــة** | |
|  |  |  | Non-Hodgkin’s lymphoma (NHL) is not contagious  سرطان الغدد الليمفاوية اللاهودجكينية هو مرض غير معدي | 1 |
|  |  |  | NHL is more common than HL  سرطان الغدد الليمفاوية اللاهودجكينية أكثر شيوعًا من سرطان الغدد الليمفاوية الهودجكينية | 2 |
|  |  |  | NHL is a hereditary disease (passed on directly from parents to children)  سرطان الغدد الليمفاوية اللاهودجكينية هو مرض وراثي | 3 |
|  |  |  | NHL can develop at all age periods; children, adults, and elderly  سرطان الغدد الليمفاوية اللاهودجكينية يمكن ان يحدث في جميع المراحل العمرية؛ الأطفال والبالغين وكبار السن | 4 |
|  |  |  | NHL stages are determined based on the number and location of lymphomas in the body  يتم تحديد مراحل سرطان الغدد الليمفاوية اللاهودجكينية بناءً على عدد وموقع سرطان الغدد الليمفاوية في الجسم | 5 |
|  |  |  | NHL most commonly appears as a solid tumor in the lymph nodes and can spread to extranodal tissues  غالبًا ما يظهر سرطان الغدد الليمفاوية اللاهودجكينية على شكل ورم صلب في الغدد الليمفاوية ويمكن أن ينتشر إلى خارج العقد | 6 |
|  |  |  | The most common symptom of NHL is a painless swelling in the neck, armpit or groin  التورم غير المؤلم في الرقبة او الإبط أو الفخذ هو العرض الأكثر شيوعا في سرطان الغدد الليمفاوية اللاهودجكينية | 7 |
|  |  |  | NHL is considered to be treatable  يعتبر سرطان الغدد الليمفاوية اللاهودجكينية قابلا للعلاج | 8 |
|  |  |  | Chemotherapy is the mainstay of treatment in patients with NHL  العلاج الكيماوي هو الركيزة الأساسية للعلاج في مرضى سرطان الغدد الليمفاوية اللاهودجكينية | 9 |
|  |  |  | Most often, the treatment of NHL is a regimen of 4 to 5 drugs known as CHOP/R-CHOP  غالبًا ما يكون علاج سرطان الغدد الليمفاوية اللاهودجكينية عبارة عن نظام دوائي يحتوي CHOP / R-CHOP من 4-5 أدوية يعرف باسم | 10 |
|  |  |  | NHL chemotherapy protocol contains oral and parenteral drugs  يحتوي العلاج الكيماوي لسرطان الغدد الليمفاوية اللاهودجكينية على أدوية تؤخذ عن طريق الفم والحقن | 11 |
|  |  |  | Radiation therapy has a limited role in the treatment of NHL relative to HL  العلاج الإشعاعي له دور محدود في معالجة الغدد الليمفاوية اللاهودجكينية مقارنة بالهودجكينية | 12 |
|  |  |  | Mothers should not breastfeed while receiving NHL chemotherapy  يجب على الأمهات عدم الإرضاع أثناء تلقي أدوية العلاج الكيماوي لسرطان الغدد الليمفاوية اللاهودجكينية | 13 |
|  |  |  | Dexamethasone drug can be used for NHL patients to prevent chemotherapy-induced nausea and vomiting  يمكن استخدام دواء ديكساميثازون لمرضى سرطان الغدد الليمفاوية اللاهودجكينية لمنع الغثيان والقيء الناجم عن العلاج الكيماوي | 14 |
|  |  |  | Anti-emetic drugs should be administered 30 to 60min before NHL chemotherapy  يجب إعطاء الأدوية المضادة للقيء قبل ثلاثين الى ستين دقيقة من العلاج الكيماوي لسرطان الغدد الليمفاوية اللاهودجكينية | 15 |

| **Section 3: Questions related to the assessment of nurses' Practice regarding NHL pharmacotherapy** | | | | | | | |
| --- | --- | --- | --- | --- | --- | --- | --- |
| N/A  لا ينطبق | Always  دائما | Often  غالبا | Sometimes  احيانا | Rarely  نادرا | Never  مطلقا | **Practice**  **الممارســــــة** | |
|  |  |  |  |  |  | I do practice to administer chemotherapy for NHL patients even before I get receiving the proper training  أمارس إعطاء العلاج الكيميائي لمرضى سرطان الغدد الليمفاوية اللاهودجكينية حتى قبل أن أتلقى التدريب المناسب | 1 |
|  |  |  |  |  |  | I do practice chemotherapy administration for NHL patients only based on my good experience  أمارس إعطاء العلاج الكيميائي لمرضى سرطان الغدد الليمفاوية اللاهودجكينية اعتمادا على خبرتي الجيدة فقط | 2 |
|  |  |  |  |  |  | When I realize that a patient has NHL, I refer him/her directly to an oncologist  عندما أدرك أن المريض مصاب بسرطان الغدد الليمفاوية اللاهودجكينية، أحيله مباشرة إلى طبيب الأورام | 3 |
|  |  |  |  |  |  | When I realize that a patient has NHL, I suggest him/her to be treated exclusively with alternative medicine  عندما أدرك أن مريضًا مصابًا بسرطان الغدد الليمفاوية اللاهودجكينية، أنصحه بالمعالجة فقط بالطب البديل | 4 |
|  |  |  |  |  |  | I don't prefer dealing with NHL patients who are experiencing serious side effects from chemotherapy  لا أفضل التعامل مع مرضى الغدد الليمفاوية اللاهودجكينية الذين يعانون من آثار جانبية خطيرة من العلاج الكيماوي | 5 |
|  |  |  |  |  |  | Before the chemotherapy administration, I make sure that NHL patient has used his/her pre-chemotherapy medications  قبل إعطاء العلاج الكيماوي، أتأكد من أن مريض الغدد الليمفاوية اللاهودجكينية قد استخدم أدوية ما قبل العلاج الكيماوي | 6 |
|  |  |  |  |  |  | I prepare chemotherapy for NHL patients in a safe and appropriate area  أقوم بتحضير العلاج الكيماوي لمرضى الغدد الليمفاوية اللاهودجكينية في منطقة آمنة ومناسبة | 7 |
|  |  |  |  |  |  | I do not eat, drink, or smoke in areas where NHL chemotherapy is administered  أنا لا آكل أو أشرب أو أدخن في مناطق إعطاء العلاج الكيماوي الخاص بالغدد الليمفاوية اللاهودجكينية | 8 |
|  |  |  |  |  |  | I wash my hands and skin thoroughly after any contact with NHL chemotherapy  أغسل يدي وبشرتي جيدًا بعد أي ملامسة للعلاج الكيماوي الخاص بالغدد الليمفاوية اللاهودجكينية | 9 |
|  |  |  |  |  |  | When I administer NHL chemotherapy, I wear personal protective tools, such as gloves & mask  عندما أقوم بإعطاء العلاج الكيماوي الخاص بالغدد الليمفاوية اللاهودجكينية، أرتدي وسائل حماية شخصية كالقفازات والكمامة | 10 |
|  |  |  |  |  |  | I receive direct instructions and follow-ups from oncologists while administering chemotherapy to NHL patients  أتلقى تعليمات ومتابعة مباشرة من أطباء الأورام أثناء إعطاء العلاج الكيميائي لمرضى سرطان الغدد الليمفاوية اللاهودجكينية | 11 |
|  |  |  |  |  |  | During administration, I change the some of supportive care medications prescribed for NHL patients (such as, antiemetics, analgesics, etc.) according to what is available at the NOC  أثناء الإعطاء، أقوم بتغيير بعض الأدوية الداعمة الموصوفة لمرضى سرطان الغدد الليمفاوية اللاهودجكينية (مثل مضادات القيء والمسكنات وما إلى ذلك) وفقًا لما هو متوفر في المركز | 12 |
|  |  |  |  |  |  | I monitor NHL patients regularly during their chemotherapy administration  أقوم بمراقبة ومتابعة مرضى الغدد الليمفاوية اللاهودجكينية بانتظام أثناء علاجهم الكيماوي | 13 |
|  |  |  |  |  |  | I support NHL patients psychologically and socially during their chemotherapy administration  أنا أدعم مرضى الغدد الليمفاوية اللاهودجكينية نفسيا ومعنويا أثناء علاجهم الكيماوي | 14 |
|  |  |  |  |  |  | I deal with chemotherapy complications for NHL patients professionally  أتعامل مع مضاعفات العلاج الكيماوي لمرضى سرطان الغدد الليمفاوية اللاهودجكينية بشكل احترافي | 15 |
| During chemotherapy administration, when I recognize a medical error in physicians' prescriptions related to NHL chemotherapy (such as, wrong dosage, or route, or regimen,..etc), I follow one of the following strategies:  أثناء اعطاء العلاج الكيماوي، عندما أدرك وجود خطأ طبي في وصفات الأطباء المتعلقة بالعلاج الكيماوي لمرضى سرطان الغدد الليمفاوية اللاهودجكينية (مثل خطأ في الجرعة، أو في طريقة الاعطاء، أو في البروتكول....إلخ) **فاني اتخذ احد التدابير التالية:** | | | | | | | 16 |
|  |  |  |  |  |  | - I keep administering medication anyway   أواصل إعطاء الدواء كما هو محدد في الوصفة |  |
|  |  |  |  |  |  | - I contact physician   اتصل بالطبيب للتأكد |  |
|  |  |  |  |  |  | - I change it to the right way   اغيره واعطيه بالطريقة الصحيحة |  |

Thank you very much. We greatly appreciate your participation
