# Supplementary material for: Evaluation of clinicians’ knowledge and practice regarding pharmacotherapy of Non-Hodgkin’s lymphoma: A multi-center study in Yemen
Source: PLoS One. 2024 Jun 5;19(6):e0304209. doi: 10.1371/journal.pone.0304209 (PMC11152296; doi:10.1371/journal.pone.0304209)
Supplement: S2 File — (DOCX) [file pone.0304209.s002.docx]

**Knowledge and practice (CP) of Physicians regarding pharmacotherapy of NHL**

This survey aims to study physicians’ knowledge and practice regarding NHL pharmacotherapy in Yemen.

| **Section 1: Demographic Data**  **البيانات الديموغرافية** | | |
| --- | --- | --- |
| **Age (Years): …………..** | **Gender:** 🞎 **Male** 🞎 **Female** | **Marital Status:** 🞎 **Single** 🞎 **Married** 🞎 **Divorced** |
| **Physicians Category:**  🞎 **Consultant** 🞎 **Specialist** 🞎 **Resident** 🞎 **GP** | | |
| **Physicians’ Subspeciality:**  🞎 **Medical Oncology** 🞎 **Radiation Oncology** 🞎 **Surgical Oncology**  🞎 **Pediatric oncology** 🞎 **Hematology** 🞎 **Other (Please Specify) …………………** | | |
| **Years of Experience in the oncology-related field:**  🞎 **less than 3 years** 🞎 **3-5 years** 🞎 **6-10 years** 🞎 **more than 10 years** | | |
| **What is your main work place where you spend most of your time?**  🞎**Office practice** 🞎**Government Hospital** 🞎**Oncology center** 🞎**Private hospital/clinic** 🞎**University teaching**  🞎 **Other (Please Specify) ………………** | | |

| **Section 2: Questions related to Physicians’ knowledge regarding NHL pharmacotherapy** | | | | |  |
| --- | --- | --- | --- | --- | --- |
| I don’t know | Disagree | Agree | **Knowledge** | |  |
|  |  |  | **Non-Hodgkin’s lymphoma (NHL) is more extranodal involvement compared to Hodgkin lymphoma (HL)** | **1** | |
|  |  |  | **The majority of NHL patients present with peripheral lymphadenopathy** | **2** | |
|  |  |  | **Immunocompromised patients are at increased risk of NHL** | **3** | |
|  |  |  | **Diffuse large B-cell lymphoma (DLBCL) is the most common aggressive type of NHL.** | **4** | |
|  |  |  | **Oral lesion is considered the main primary diagnostic factor of non-Hodgkin's lymphoma** | **5** | |
|  |  |  | **The goal of treatment for patients diagnosed with aggressive NHL is cure** | **6** | |
|  |  |  | **The effective chemotherapy regimen for aggressive NHL is often complex, involving more than one agent** | **7** | |
|  |  |  | **Radiation therapy has a limited role in NHL relative to HL since NHL is more often a systemic disease** | **8** | |
|  |  |  | **Patients with NHL of the central nervous system have a poor response to chemotherapy compared to those with other forms of NHL** | **9** | |
|  |  |  | **Dosage adjustment for NHL patients based on body surface area and organ dysfunction (renal/hepatic impairment)** | **10** | |
|  |  |  | **Rituximab-containing regimen may reactivate latent diseases, such as hepatitis B** | **11** | |
|  |  |  | **Hyper-CVAD and EPOCH regimens are recommended for patients with HIV-related lymphoma** | **12** | |
|  |  |  | **R-CHOP is the first line therapy for DLBCL with bulky and nonbulky stages** | **13** | |
|  |  |  | **ESHAP and DHAP are the more commonly salvage regimens used in patients with relapsed or refractory NHL** | **14** | |
|  |  |  | **Dexamethasone can be used to prevent acute and delayed CINV for moderately and highly emetogenic chemotherapy** | **15** | |
|  |  |  | **Mesna should be used to prevent cyclophosphamide-induced hemorrhagic cystitis** | **16** | |
|  |  |  | **Tumor lysis syndrome should be assessed during NHL treatment to prevent AKI** | **17** | |
|  |  |  | **Bleomycin-containing regimens may induce pulmonary fibrosis in NHL patients** | **18** | |

| **Section 3: Questions related to Physicians’ Practice regarding NHL pharmacotherapy** | |
| --- | --- |
| **Practice** | |
| **In the past 5 years, in which of the following training activities regarding NHL therapy** **have you participated?**  🞎 Medical conferences  🞎 Scientific meetings  🞎 Postgraduate medical training (e.g., residency, fellowship)  🞎 Medical Research   🞎 Medical Websites and Programs  🞎 Other (Please Specify) …………………  🞎 None | **1** |
| **Are you currently involved with teaching medical students and/or residents?**  🞎 No 🞎 Yes | **2** |
| **What type of medical records system do you use during your practice?**  🞎 Paper records and charts  🞎 Partial electronic medical records  (e.g., lab results available electronically, but patient history on paper)  🞎 In transition from paper to full electronic medical records  🞎 Full electronic medical records | **3** |
| **How often do you use updated guidelines for treating NHL patients?**  🞎 Never  🞎 Rarely  🞎 Sometimes  🞎 Often  🞎 Always  🞎 N/A | 4 |
| **Before prescribing chemotherapy medications for NHL patients, how often do you consider the potential drug-drug interactions and contraindications?**  🞎 Never  🞎 Rarely  🞎 Sometimes  🞎 Often  🞎 Always  🞎 N/A | **5** |
| **How often do you prescribe off-label and/or unlicensed drugs for NHL patients?**  🞎 Never  🞎 Rarely  🞎 Sometimes  🞎 Often  🞎 Always  🞎 N/A | 6 |
| **In your practice, how often do you change the chemotherapy protocol based on the desire of NHL patients?**  🞎 Never  🞎 Rarely  🞎 Sometimes  🞎 Often  🞎 Always  🞎 N/A | 7 |
| **In your practice, how often do you encounter that you are unable to order appropriate tests or treatments for NHL patients because of their cost?**  🞎 Never  🞎 Rarely  🞎 Sometimes  🞎 Often  🞎 Always  🞎 N/A | 8 |
| **In your prescription, how often have you substituted chemotherapy medications for NHL patients due to their unavailability?**  🞎 Never  🞎 Rarely  🞎 Sometimes  🞎 Often  🞎 Always  🞎 N/A | **9** |
| **How often do you perform surveillance and screening tests for a NHL survivor who is currently asymptomatic?**  🞎 Every 6 months  🞎 Yearly  🞎 Only If indicated  🞎 Never  🞎 I don't Know  🞎 Other (please specify)…………………… | **10** |
| **How often do you practice counseling for your NHL patients?**  🞎 Never  🞎 Rarely  🞎 Sometimes  🞎 Often  🞎 Always  🞎 N/A | **11** |
| **During your practice, how often have you encountered serious side effects for your NHL patients?**  🞎 Never  🞎 Rarely  🞎 Sometimes  🞎 Often  🞎 Always  🞎 N/A | **12** |
| **How often do you provide nurses and/or other health care practitioners with a comprehensive summary including detailed information about NHL treatment?**  🞎 Never  🞎 Rarely  🞎 Sometimes  🞎 Often  🞎 Always  🞎 N/A | **13** |
| **How often do you** **closely supervise nurses and/or other health care practitioners while they are administering chemotherapy to patients with NHL to ensure proper and correct administration?**  🞎 Never  🞎 Rarely  🞎 Sometimes  🞎 Often  🞎 Always  🞎 N/A | **14** |
| **How often do you follow-up directly NHL patients while they are receiving chemotherapy to avoid any adverse effects or complications that can occur for patients?**  🞎 Never  🞎 Rarely  🞎 Sometimes  🞎 Often  🞎 Always  🞎 N/A | **15** |
| **What is the main problem you encounter during your practice that might significantly affect the treatment outcomes of NHL patients?** You can choose more than one option  🞎 Side effects of chemotherapy  🞎 Patients' noncompliance/ignorance  🞎 Unreliable diagnostic tests results  🞎 Inappropriate administration of chemotherapy  🞎 Unavailability of chemotherapy medications  🞎 High cost of medications and diagnostic lab tests  🞎 The psychological and economic condition of the patients  🞎 Other (Please Specify) …………………  🞎 None | **16** |

**Thank you very much. We greatly appreciate your participation**
